# HOME-BASED END-OF-LIFE CARE COOPERATION BETWEEN VISITING NURSES AND CARE MANAGERS IN JAPAN

**DOI:** 10.1093/geroni/igad104.3394

**Published:** 2023-12-21

**Authors:** Keiko Ono

**Affiliations:** Aomori University of Health and Welfare, Aomori, Aomori, Japan

## Abstract

End-of-life care patients use home-visit nursing services under Japanese long-term care insurance; however, they require a care manager to implement the care plan. However, the cooperation procedure of home-based medical care between visiting nurses and care managers has not been adequately researched. Thus, this study aimed to clarify the presence of support between visiting nurses and care managers to be adapted to this situation. This study was conducted in March and April 2023 at home-visit nursing stations located in a prefecture in Japan. Self-administered questionnaires were mailed to 192 visiting nurses in Japan, and 136 response forms (70.8%) were received through postal mails. A total of 130 questionnaires (67.7%) were determined to be valid responses, which also included the participants’ written consent for research. The presence or absence of work experience and the importance of collaboration in the preparatory, introductory, stable, and near-death stages of end-of-life care were answered. The presence of work experience at each stage was over 53.1%. The importance of collaboration at each stage was over 76.9%. Query regarding collaborating well with care managers was answered on a scale from 0 to 100 %. Good collaboration with care managers had an average score of 75.4%. The presence or absence of experience and the importance of collaboration items were clarified at each stage. The research findings could contribute to the development of education and training. This work was supported by JSPS KAKENHI Grant Number JP21K02802.

